# Bioinformatic identification of previously unrecognized amyloidogenic proteins

**DOI:** 10.1016/j.jbc.2022.101920

**Published:** 2022-04-09

**Authors:** Gregory M. Rosenberg, Kevin A. Murray, Lukasz Salwinski, Michael P. Hughes, Romany Abskharon, David S. Eisenberg

**Affiliations:** 1 Departments of Chemistry and Biochemistry and Biological Chemistry, UCLA-DOE Institute, Molecular Biology Institute, and Howard Hughes Medical Institute, UCLA, Los Angeles, California, USA; 2 Department of Cell and Molecular Biology, St Jude Children’s Research Hospital, Memphis, Tennessee, USA

**Keywords:** amyloid, protein structure, Charcot–Marie–Tooth disease electron microscopy, intrinsically disordered protein, low-complexity domain, LCD, low-complexity domain, NIH, National Institutes of Health, TFG, TRK-fused gene protein, ThT, thioflavin T

## Abstract

Low-complexity domains (LCDs) of proteins have been shown to self-associate, and pathogenic mutations within these domains often drive the proteins into amyloid aggregation associated with disease. These domains may be especially susceptible to amyloidogenic mutations because they are commonly intrinsically disordered and function in self-association. The question therefore arises whether a search for pathogenic mutations in LCDs of the human proteome can lead to identification of other proteins associated with amyloid disease. Here, we take a computational approach to identify documented pathogenic mutations within LCDs that may favor amyloid formation. Using this approach, we identify numerous known amyloidogenic mutations, including several such mutations within proteins previously unidentified as amyloidogenic. Among the latter group, we focus on two mutations within the TRK-fused gene protein (TFG), known to play roles in protein secretion and innate immunity, which are associated with two different peripheral neuropathies. We show that both mutations increase the propensity of TFG to form amyloid fibrils. We therefore conclude that TFG is a novel amyloid protein and propose that the diseases associated with its mutant forms may be amyloidoses.

Low-complexity domains (LCDs) are common, but functionally mysterious regions of proteins in the human proteome, of which several are associated with amyloidoses (1, 2, 3). LCDs are characterized by long segments made up of relatively low sequence diversity and are also commonly intrinsically disordered. LCDs are thought to be integral to the self-association of some proteins involved in RNA binding, the formation of membraneless organelles, and the self-association of intermediate filament proteins (4, 5, 6, 7, 8, 9), but not all proteins with LCDs exhibit these functions. Whereas, subsequent dissociation of these complexes is a hallmark of the normal function of LCDs, proteins with LCDs may become prone to aggregate irreversibly into pathogenic amyloids because of missense mutations, which encourage protein misfolding (3, 10, 11). Some examples of amyloidogenic LCD-containing proteins are FUS, TDP43, and HNRNPA1, which are all associated with ALS.

Because LCDs are often disordered, cross-β structures are present in LCD condensates (12), and many proteins containing them form amyloids in disease; we speculate that these domains may be more susceptible to mutations that cause the formation of an amyloid to be energetically favorable. Under this assumption, we chose to focus our search for novel amyloidogenic proteins on those proteins that contain an LCD (Fig. 1). This search for unidentified amyloidogenic proteins based on pathogenic mutations expands on previous work (9, 13) by considering a larger subset of the human proteome. Our approach is agnostic to details about the queried proteins (besides identifying LCDs based on amino acid sequence) such as their functions or the diseases with which they are associated. Also, while our approach does identify many known amyloidogenic proteins, our focus is solely on those that have never been documented to form amyloid fibers either *in vivo* or *in vitro*.

**Figure 1:**
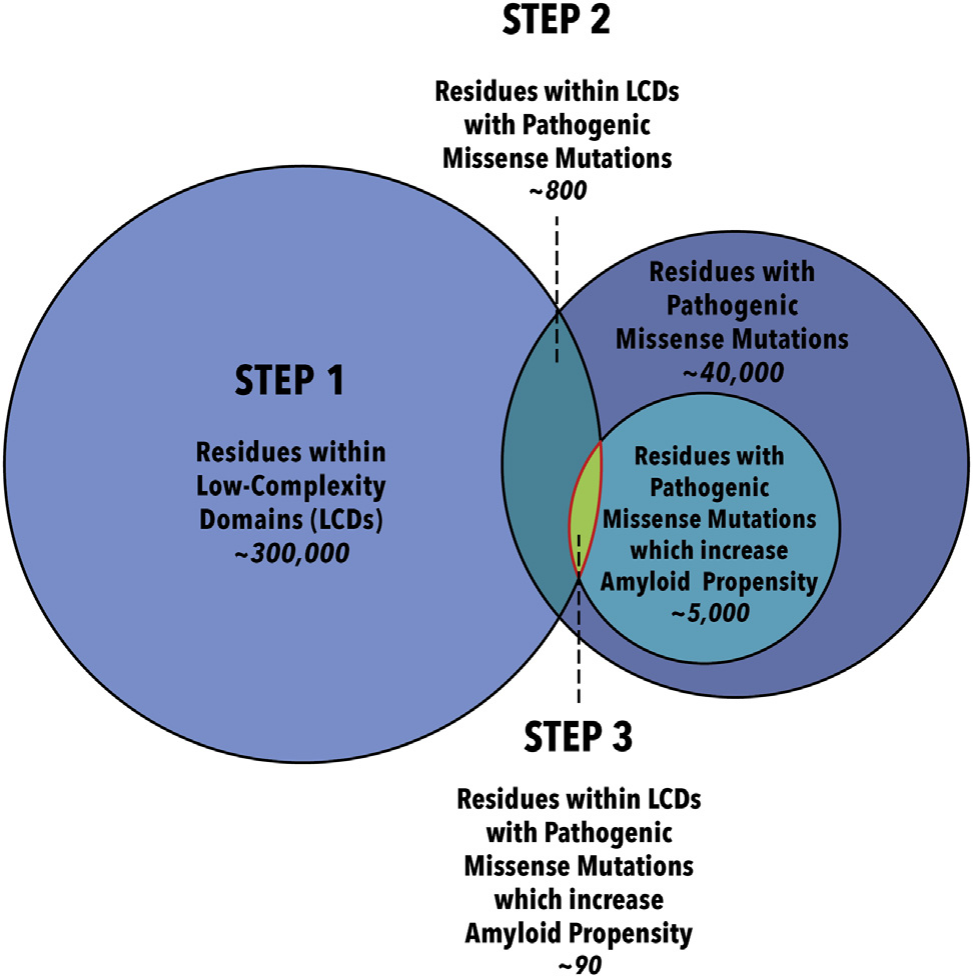
**Schematic representation of our algorithm for identification of previously unrecognized amyloidogenic proteins.** These proteins contain the mutated residues in the set at the intersection of all three circles: residues within LCDs with pathogenic missense mutations that increase amyloid propensity. We determined these residues in the three steps shown. Estimates for the number of residues represented in steps 1 to 3 are derived from this study. To estimate the number of residues with pathogenic missense mutations, we used Simple ClinVar (https://simple-clinvar.broadinstitute.org/). To calculate the estimate for the number of missense mutations that increase amyloid propensity, we extrapolated the percentage of mutations within LCDs that increase amyloid propensity (88/732; ∼12%) to our estimate of total known pathogenic missense mutations in humans (∼40,000) and rounded up. LCD, low-complexity domain.

Here, we advance computational screening methods to identify mutations that may cause a functional LCD to become amyloidogenic (9). We define an amyloid as an irreversible fibrous protein aggregate with a cross-β-sheet scaffold. The common methods of experimental identification of amyloid are the binding of amyloidophilic dyes such as Congo red or thioflavin T (ThT) and X-ray diffraction revealing the ∼4.7 to 4.8 Å separation of β-strands and ∼10 Å separation of β-sheets. Amyloids are found in a wide range of diseases from Alzheimer’s to type II diabetes (14). These amyloidoses are characterized by deposition of insoluble amyloid aggregates that, by mechanisms not completely understood, lead to cellular injury, tissue damage, and organ dysfunction (15). If amyloid deposition drives disease, it is crucial to identify the protein responsible to develop reliable treatments for the disease. We propose that many diseases have yet undiscovered amyloid components to their etiology. In this work, our algorithm identifies numerous known amyloidogenic mutations as well as many mutations not previously associated with any amyloidoses. Among the second group of mutations, we demonstrate that two from the protein TRK-fused gene protein (TFG) increase the amyloid propensity of the protein. The identification of this and other potential undiscovered amyloid proteins is important for understanding the pathogenesis and expanding the treatment options of their associated diseases.

## Results

To identify LCDs, we first applied the SEG algorithm (16) to the human proteome to categorize amino acid segments as either high complexity or low complexity. We then conservatively defined an LCD as any low-complexity segment of at least 35 amino acids with leeway for five interrupting high-complexity amino acids in a row. Under these criteria, 3251 human proteins contain at least one LCD. We then scoured UniProt Knowledgebase (UniProtKB) (17), Online Mendelian Inheritance in Man (OMIM) (18), and ClinVar (https://simple-clinvar.broadinstitute.org/) (19) for pathogenic missense mutations within the LCDs of these proteins and found 738 documented disease-related mutations. This set of mutations was collected while remaining agnostic to the functional consequences for the affected protein, so among these mutations are not only some that are pathogenic because they increase amyloid propensity but also many that are pathogenic for various other reasons unrelated to amyloidogenicity.

**Figure 2:**
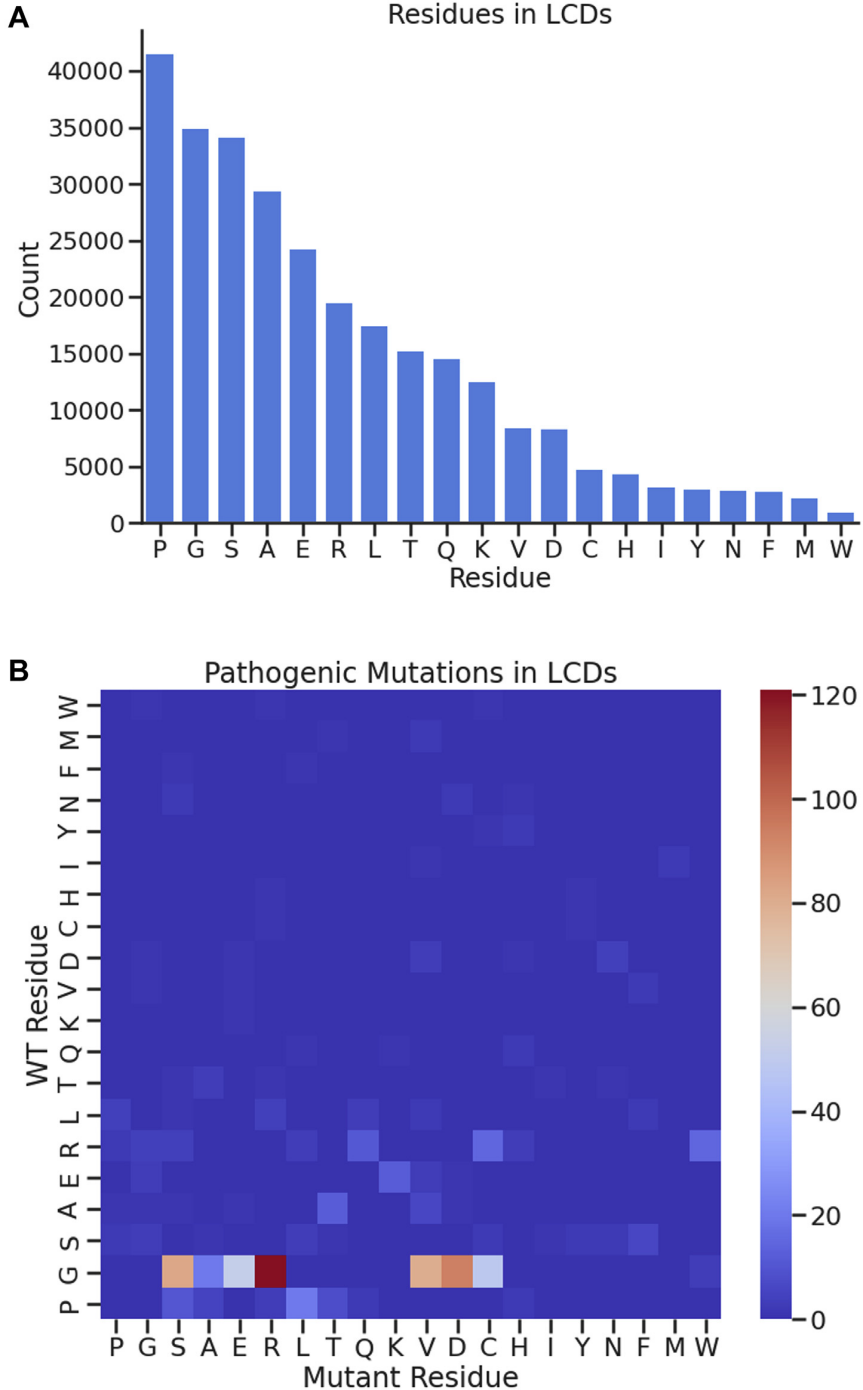
**Census of amino acid residues present in LCDs.***A*, counts of all residues in LCDs in the human proteome. *B*, heatmap displaying counts of all LCD residues involved in documented pathogenic missense mutations and which residues they change into. Note that many residue changes are not possible from single-nucleotide variants, which accounts for many of the data points of 0 in the heatmap. LCD, low-complexity domain.

Among the residues that make up LCDs, prolines are the most common, followed by glycine, serine, and alanine (Fig. 2*A*). Glycine is by far the most common residue to be replaced in pathologies, with 501 of the 738 disease-related mutations being changed from glycine (Fig. 2*B*). The most common mutation is from glycine to arginine, followed in descending order by glycine to aspartate, glycine to valine, and glycine to serine. The next most common residue to be replaced in pathologies is arginine with 57 mutations. This is followed by proline mutations, making up 50 of the documented disease-related mutations. These findings suggest that glycine residues are especially important in maintaining normal function of human LCDs.

**Figure 3:**
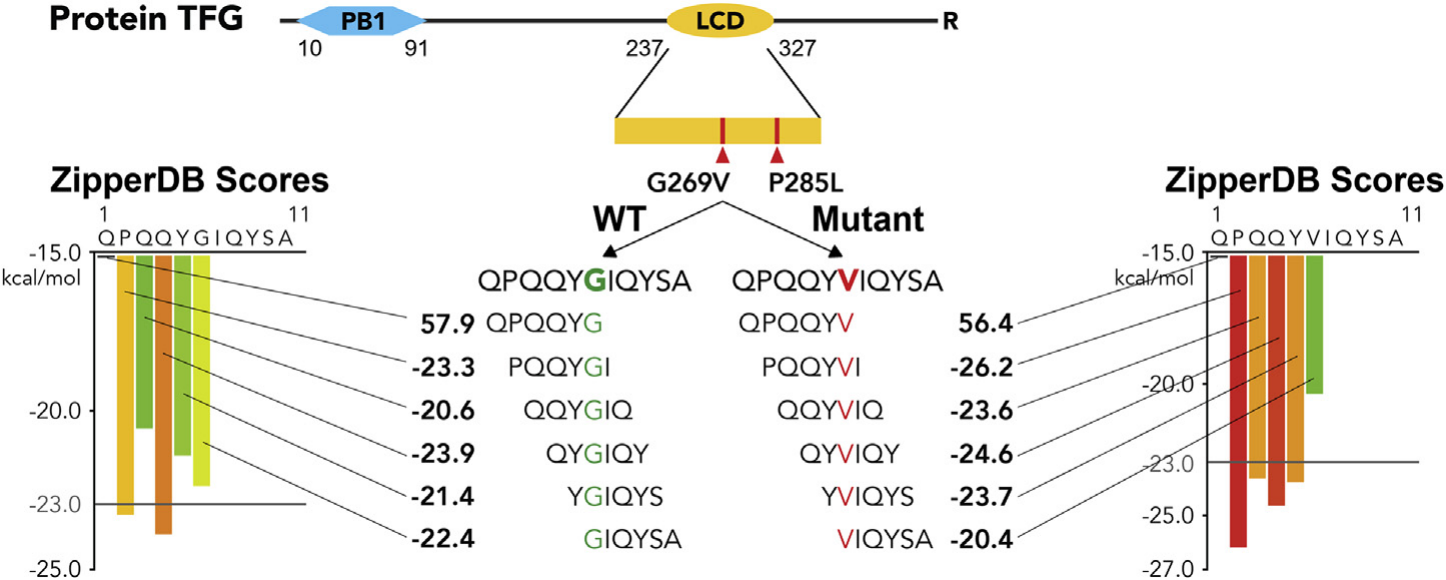
**Schematic summary of the methodology by which we discovered amyloidogenic mutations.***Top*, diagram of protein TFG that contains a PB1 domain and a low-complexity domain. We investigated only mutations in the low-complexity domain. *Bottom*, analyzing the sequence context of the mutant residue. We calculated Rosetta energy scores using ZipperDB for every hexamer containing the WT residue as well as the mutant residue. Each WT hexamer is compared with its corresponding mutant hexamer. WT scores greater than −23.0 that correspond to a mutant score less than −23.0 imply greater amyloid propensity and are of the most interest. TFG, TRK-fused gene protein.

**Figure 4:**
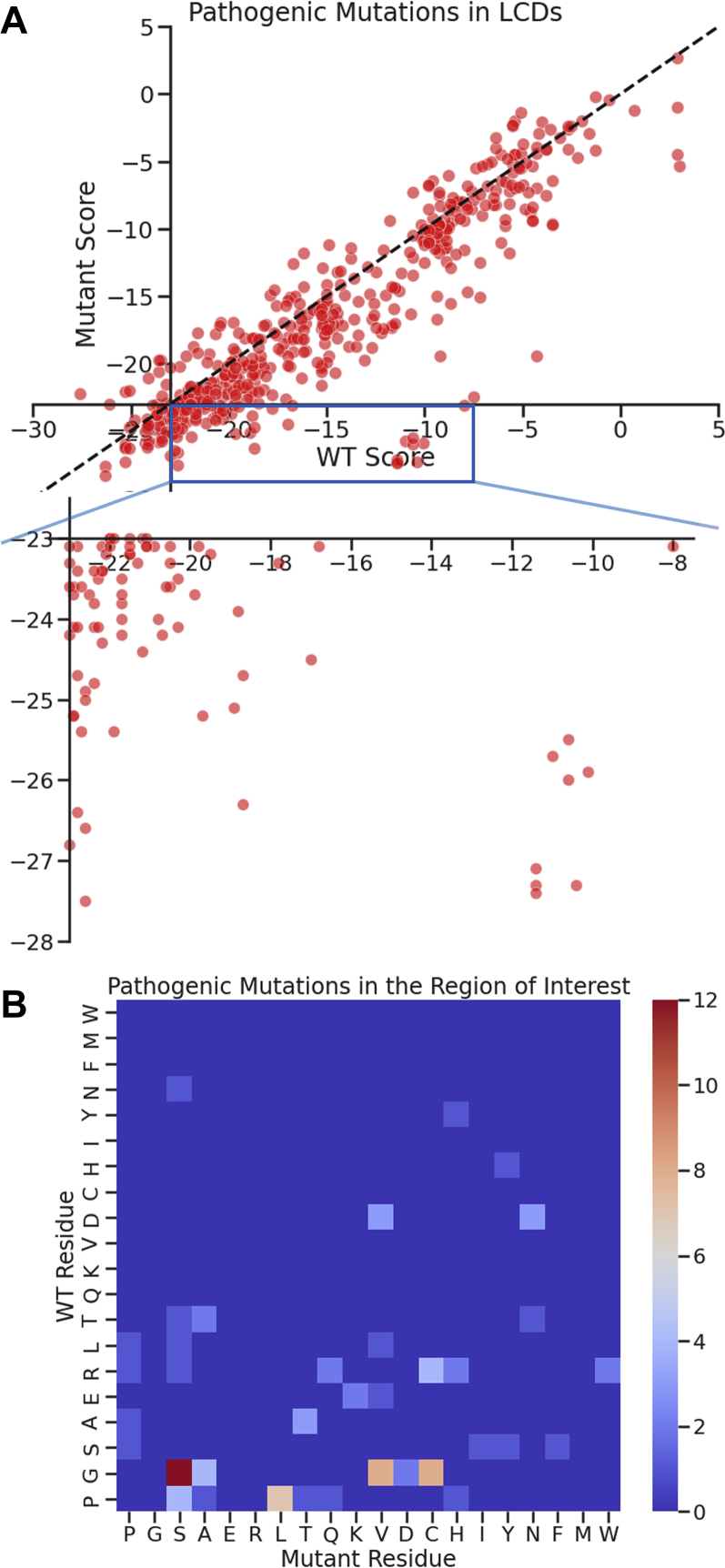
**Mutant residues in the LCDs throughout the human proteome with greater propensity to form amyloid than their corresponding WT residue.***A*, energy scores of WT and mutant segments in LCDs computed by ZipperDB. Because each mutation generates six possible score pairs, only the score pair that mapped to the “region of interest” (*inset*) or with the greatest negative change from WT to mutant score is plotted for each mutation. The *dashed line* shows mutations that do not affect the ZipperDB score. The *x* and *y* intercepts are both at −23.0 kcal/mol of the segment, the ZipperDB threshold for a predicted amyloid-forming steric zipper. *Inset* contains a zoomed view of the lower right quadrant of the plot that is the “region of interest” containing points corresponding to a WT segment with a score above −23.0 kcal/mol of segment and a mutant segment with a score below −23.0 kcal/mol of segment, indicating a mutation that increases the amyloid propensity. *B*, heatmap displaying counts of the kinds of mutational changes in the “region of interest.” LCD, low-complexity domain.

Next, we sought to identify the mutations from the set of 738 pathogenic mutations that increase the propensity of a functional sequence to form a steric zipper, the common adhesive protein motif driving amyloid (20). To achieve this, we used ZipperDB, a database that predicts the fibril-forming propensity of segments within proteins (21). ZipperDB evaluates the energetic fit of 6-residue segments in the conformation of a steric zipper. Therefore, for each mutation, we analyzed two 11-residue sequences centered on the mutated residue: one containing the WT residue and one containing the mutant residue. For each of these sequences, all six possible hexamers containing the residue of interest were assigned energy values by ZipperDB (Figs. 3 and 4*A*). Proline residues are not energetically favorable in β-sheets, so segments containing this residue tend to have very high positive energy scores. Since proline is a common residue in LCDs (Fig. 2*A*), this skews a significant portion of the data to high positive values, and Figure 4*A* does not include this skewed portion of the plot (Fig. S1).

In detail, we identified amyloidogenic mutations as follows: we predict a mutation to be amyloidogenic if a hexamer containing the WT residue has an energy value greater than the ZipperDB threshold of −23 kcal/mol (therefore with lower amyloid propensity) and the corresponding hexamer containing the mutant residue has an energy value lower than or equal to −23 kcal/mol (therefore with higher amyloid propensity) (Figs. 3, 4*A* and Table S1). Mutations that generated these hexamers were predicted to cause a gain-of-function amyloid propensity. This group contains 88 mutations, the most common changes being glycine to serine, glycine to valine, glycine to cysteine, and proline to leucine (Fig. 4*B*).

Conspicuously, changes to charged amino acids are greatly underrepresented in this predicted-amyloidogenic set. For example, glycine to arginine changes are completely absent, whereas they are the most common type of pathogenic mutation in LCDs in general. This is unsurprising, however, since amino acid side chains on the interior of a steric zipper need to be able to pack closely together as well as to stack on top of each other along the fiber axis, and these charged side chains will repel each other making amyloid formation energetically unfavorable.

Furthermore, statistical analysis reveals that glycine is the only residue that, when mutated, has statistically significant differences in whether the mutation is predicted to be amyloidogenic or not depending on which residue it mutates into (Table S2). In other words, for glycine only, the amino acid it mutates into is significant in determining whether the mutation is predicted to be amyloidogenic or not. Taken together, there are many documented mutations within LCDs that may drive a functional sequence to become amyloidogenic, but mutations from glycine and proline are more likely than others to be of this kind.

To validate that our approach is capable of identifying amyloidogenic mutations, we combed the list for known amyloidogenic mutations and found several. Two of the listed mutations in hnRNPA1 (D314V and D314N) and the mutation in hnRNPA2B1 (D302V) have been experimentally shown to induce fiber formation (3). One of the listed mutations in KRT8 (G62C) has been demonstrated to enhance aggregation propensity (9). Desmin, a protein that can form amyloid fibers in myofibrillar myopathy (22), has three mutations in the list (S2I, S46F, and S46Y) that are associated with myofibrillar myopathy and has been shown to cause abnormal aggregation (7). A mutation in PABPN1 (G12A) mimics a pathogenic polyalanine expansion (23), and an extended polyalanine segment in this protein has been shown to induce fiber formation (24). Known amyloidogenic proteins TDP43 and FUS also have mutations that appear on the list, but so far, these mutations have not been experimentally tested for increased amyloid propensity. These examples confirm that our approach can identify at least some mutations that contribute to the formation of amyloid fibers.

To determine if our approach identifies novel amyloid mutations, we analyzed one of the pathogenically altered proteins, TFG. As part of its native function, TFG self-associates into octameric oligomers, and its LCD facilitates these octamers to form larger complexes (25). The two mutations in TFG that were identified by our method, G269V and P285L, have been associated with Charcot–Marie–Tooth disease type 2 and hereditary motor and sensory neuropathy with proximal dominant involvement, respectively (26, 27), and both mutations were shown to result in abnormal aggregation of the protein. We also found that the WT protein and the protein with the P285L mutation are able to phase separate *in vitro* in the presence of a crowding agent, but with the G269V mutation, the protein forms amorphous aggregates (Fig. S2).

**Figure 5:**
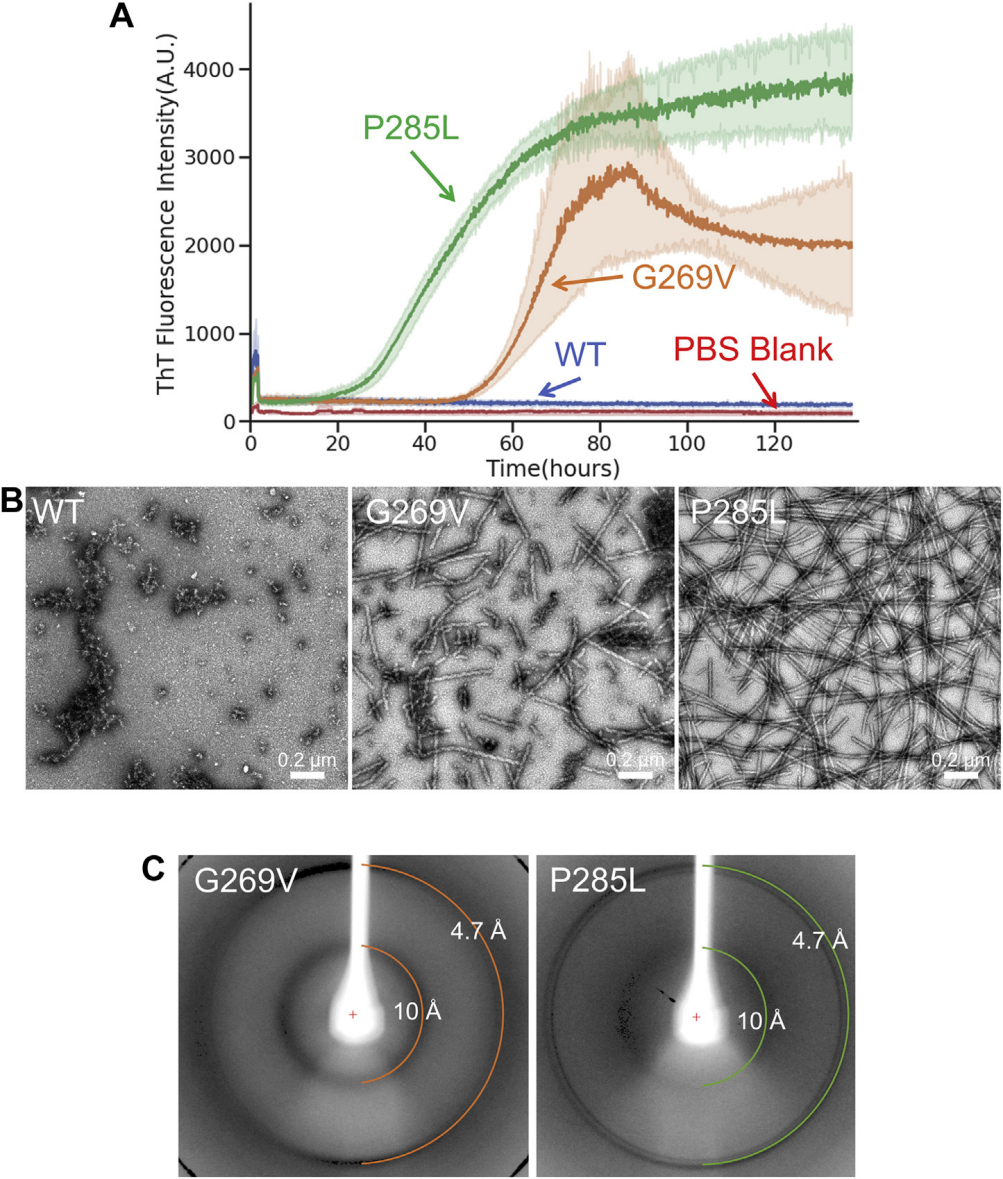
**Amyloid properties of the LCD of protein TFG.***A*, time-dependent ThT fluorescence for TFG LCD mutants. G269V and P285L are documented pathogenic mutations of TFG. All constructs are at 50 μM concentration in PBS with ThT at 40 μM concentration. Each construct has n = 6 technical replicates, except the PBS blank that has n = 3 technical replicates, and *y*-axis values represent the mean ThT fluorescence value of all replicates for each construct. *B*, electron micrographs of the samples at the end point of the ThT curves. Fibers were present only in the mutant constructs. *C*, X-ray fiber diffraction of TFG fibers. Rings are present at 4.7 and 10 Å spacing with distinct wedges, indicative of cross-β structure. LCD, low-complexity domain; TFG, TRK-fused gene protein; ThT, thioflavin T.

We expressed and purified the LCD of TFG (residues 237–327), fused with mCherry to increase solubility, in three forms: the WT sequence, containing the G269V mutation (G269V), and containing the P285L mutation (P285L). Each construct was shaken at 37 °C for 138 h with ThT, a dye that fluoresces in the presence of amyloid fibers (28, 29). Both mutant constructs demonstrated strong ThT fluorescence, whereas the WT construct did not (Fig. 5*A*). The presence of fibers from the mutant constructs and the absence of fibers from the WT construct were confirmed by electron microscopy (Fig. 5*B*). Both mutant fibers displayed an apparent twist, typical of amyloid fibers.

The amyloid nature of the TFG 237 to 327 fibers was also confirmed through X-ray diffraction. Drops of solutions containing the fibers were suspended between glass rods and allowed to dry, which aligns the fibers between the rods. The fibers were placed in an X-ray beam with the length of the fibers perpendicular to the direction of the beam. The resulting diffraction pattern for both mutant fibers displayed distinct rings at resolutions representing the characteristic dimensions of an amyloid fiber: 10 Å representing the intersheet spacing and 4.7 Å representing the interstrand spacing (Fig. 5*C*). We observed nearly the same behavior in the full-length protein, with the differences being faster fiber formation, likely because of lower solubility, and the WT sequence was able to form fibers, although at a significantly slower rate (Fig. S3). In short, we found TFG exhibits amyloid behavior when pathogenically altered, as predicted by our bioinformatic approach.

## Discussion

### Interpretation of predicted amyloid-driving mutations in terms of atomic structure

The most common residues within LCDs to be replaced in pathologies are glycine, arginine, and proline, all of which are known to be important for the normal function of many LCDs, especially with regard to the regulation of phase-separation properties (30, 31, 32). Because of their functional importance in LCDs, altering these residues can result in pathology for various reasons unrelated to amyloidogenesis, but in this study, we are most interested in pathogenic mutations in LCDs that result in increased amyloidogenicity specifically, and the trends in predicted amyloidogenic mutations in LCDs are slightly different than overall pathogenic mutations in LCDs, with proline being the second most common residue to have mutations predicted to be amyloidogenic rather than arginine.

The observed frequency of predicted amyloid-driving mutations (Fig. 4*B*) can to some extent be understood in terms of atomic structures. Mutational replacement of a residue can destabilize the native state, favoring a conformational change leading to a pathogenic loss of native function; alternatively, replacement can favor formation of amyloid, leading to pathogenic amyloid. Both effects are possible with replacement of glycine, which we identified as the most commonly replaced residue associated with disease (Fig. 2*B*) and with the most replacements predicted to be amyloidogenic (Fig. 4*B*). Glycine residues confer flexibility to the peptide backbone, which is important in maintaining the liquid properties of phase-separated protein droplets (30). Glycine's lack of a side chain larger than a hydrogen atom disfavors secondary structure, since it grants an extra flexible peptide bond, and hence does not preferentially form α-helices or β-sheets (33). This means that glycine residues are less likely than other residues to contribute to the stability of an amyloid fiber core that is typically enriched with β-sheets. We previously demonstrated that glycines can introduce kinks into the backbone of β-strands in amyloid structures derived from low-complexity segments (34), which may partially destabilize kinked backbones in low-complexity amyloid structures. We have also found that glycines lead to extended β-strand motifs in these low-complexity amyloid structures, which may also contribute to their lability (35). Considering only single-nucleotide variant missense mutations, glycine can potentially mutate into serine, alanine, glutamic acid, arginine, valine, aspartic acid, cysteine, and tryptophan (Fig. S4). The most common glycine mutations that we predicted to be amyloidogenic are glycine to valine, glycine to cysteine, and glycine to serine.

Glycine to valine is the second most common glycine mutation predicted to be amyloidogenic. Valine has a relatively bulky side chain, which is branched at the β carbon, and side chains like this prefer to form β-sheet secondary structures. Changes from glycine to valine would likely facilitate the formation of a steric zipper for this reason, especially if the surrounding sequence is already somewhat amyloid prone other than being broken by the glycine residue and, as is the case for some glycine-rich LCD proteins (FUS and TDP43), forms metastable complexes with copies of itself as part of its function. One of the TFG mutations that increases amyloid propensity is a glycine to valine mutation (G269V).

Glycine to cysteine is the third most common glycine mutation, which is predicted to induce amyloid propensity. The cysteine side chain is a thiol group that, when oxidized, can form disulfide bonds with other oxidized cysteine side chains. These covalent bonds normally greatly increase the stability of globular proteins, but these are normally intramolecular bonds. Intermolecular disulfide bonds can potentially stabilize cross-β interactions and contribute to the formation of amyloid fibers (36, 37). This intermolecular disulfide bonding is especially likely if the glycine to cysteine mutation creates a sequence with only a single cysteine in a region that is routinely exposed to copies of itself, as in many LCDs in which cysteines are not particularly common (Fig. 2*A*). A glycine to cysteine mutation in KRT8 (G62C) has been shown to increase amyloid propensity (9).

Glycine to serine is the most common residue change out of the glycine mutations, which are predicted to increase amyloid propensity. It is unclear exactly what benefit a serine residue would bring to the structure of an amyloid fiber since it has a polar side chain that is not able to form hydrogen bonds with itself when stacked like glutamine side chains. Nonpolar residues are more common on the interior of pathogenic amyloids, but polar residues do sometimes exist in steric zippers. One alternative role for serine is possibly facilitating the formation of a turn and contributing to the dagger-like fold, which is seen in some structures of amyloid cores (20). Glycine to serine mutations in FUS, which are associated with ALS (G206S and G191S), appear in our list of potentially amyloidogenic mutations.

After glycine mutations, proline mutations are the most common mutations predicted to be amyloidogenic in our set of interest (Fig. 4*B*). Like glycine, proline is thought of as a secondary structure breaker. Proline side chains break up β-strands because of steric restrictions of their phi and psi angles imposed by their unique side chains, which forms a bond with the α-amino nitrogen of the peptide backbone (38). Besides breaking the secondary structure necessary to form a steric zipper, the proline side chain also disfavors the formation of amyloid fibrils by removing the peptide backbone nitrogen’s availability for interstrand hydrogen bonding, which normally is a major contributor to the stability of the overall fiber (7). With only single-nucleotide variant missense mutations, proline can potentially mutate into serine, alanine, arginine, leucine, threonine, glutamine, and histidine (Fig. S4).

The most common proline mutation predicted to be amyloidogenic is from proline to leucine. Leucine is a nonpolar side chain with a branched gamma carbon. Packing of hydrophobic residues in the core of an amyloid fiber tends to increase stability and is preferred to polar residues in pathogenic amyloids (20). This explains how a mutation to leucine could contribute to the formation of an amyloid fiber. One of the TFG mutations that increases amyloid propensity is a proline to leucine mutation (P285L).

It is important to consider the possibility that the distribution of common mutations predicted to be amyloidogenic may be sequence context dependent rather than a result of which residue changes are most amyloidogenic in general. In other words, the reduced amino acid diversity in individual LCDs may limit which mutations actually increase the likelihood of a steric zipper within their sequence context. This could explain phenomena like proline to leucine mutations being more common in the list than proline to threonine mutations, the latter of which would have comparatively higher β-sheet propensity yet is much less common. Another consideration is codon limitations. Single-nucleotide missense mutations only allow for a limited number of amino acid changes and some are more likely than others because of similarities in codons and codon number (Fig. S4). This explains why proline to leucine mutations are the most common type of proline mutation among the mutations predicted to be amyloidogenic, but there are no glycine to leucine mutations, since glycine to leucine mutations are impossible with only a single-nucleotide change. It also makes it more significant that glycine to cysteine mutations are commonly predicted to be amyloidogenic even though there are fewer ways for single-nucleotide changes to result in that mutation compared with glycine to alanine mutations (Fig. S4). The underlying reasons for the distribution of mutations in this list require further study.

### Use of ZipperDB to assess mutations most likely to be amyloidogenic

We used ZipperDB to score sequences on their propensity to form a steric zipper, the core of amyloid fibers. ZipperDB threads sequences onto a peptide backbone based on the crystal structure of NNQQNY, a fibril-forming peptide from the yeast sup35 prion protein and generates an energy score. It is possible to utilize a different peptide backbone for steric zipper predictions, but NNQQNY is the default and the one used for all the existing segments in the database. ZipperDB is not the only existing method for predicting amyloid fibers, but it is useful for high-throughput applications and is structure based rather than sequence based. Different amyloid-prediction tools can be variable in their predictions. To demonstrate this, we used AMYLPRED2 (http://thalis.biol.uoa.gr/AMYLPRED2/) (39) to predict amyloidogenic regions in TFG and its mutants (Fig. S5). AMYLPRED2 employs up to 11 different amyloid-prediction methods and outputs their consensus. We ran AMYLPRED2 using 10 methods, and there was enough consensus for a high-confidence prediction of an amyloid segment containing the G269V mutation but not the P285L mutation. The scoring system of ZipperDB is calibrated against experimental amyloid structures and has proven very reliable in predicting sequences that can form fibers (3, 21, 40, 41). Though, there are some important drawbacks to note. ZipperDB only considers homozippers, whereas many amyloid fibers contain heterozipper interfaces at their cores, which can contribute to underprediction of amyloid-forming segments. Also, ZipperDB does not consider the sequence context of each segment it analyzes, so segments that may form fibers in theory may not actually be able to interact because of being buried in the interior of the protein or some other interference from surrounding segments, which can contribute to overprediction of amyloid-forming segments. These considerations mean that our method of identification has the potential to miss amyloids that would have been better identified by other methods and also include erroneous amyloid predictions.

### Validation of our algorithm to identify amyloidogenic proteins

Our analysis has proven able to identify mutations that grant amyloidogenic gain of function. Within the list of mutations predicted to be amyloidogenic, there were many that have been previously demonstrated to promote amyloid fibrillation as well as many mutations that have unknown structural consequences. Also, there were many mutations in proteins known to be able to form amyloids, but the mutations themselves have no documentation on their biochemical consequences. It is also important to note that even if the mutation has the potential to cause the protein to form an amyloid, this does not necessarily mean that the protein will form an amyloid under physiological conditions in disease. This makes it difficult to gauge the specificity and sensitivity of our method. These considerations also factored into the model protein we used to validate our method, TFG, since TFG had previously been shown to aggregate when pathogenically altered in both cell models and tissue biopsies from diseased patients. However, other proteins in our list have also been demonstrated to aggregate when pathogenically altered but have not been shown to be amyloids, namely LMNA and CHCHD10 (42, 43). We are currently analyzing the behavior of these proteins with regard to amyloid formation. Some other interesting proteins from our list of potential amyloid mutations include proteins that have the Gene Ontology molecular function term “identical protein binding.” This term encompasses not only all the known amyloid proteins from the list along with TFG and LMNA but also other interesting proteins such as UBQLN2, which is involved in some forms of ALS (44), and GRM6, in which mutations can lead to night blindness because of disrupted trafficking of the protein (45).

The LCD of TFG was able to form amyloid fibers only when containing mutations, in line with what our method predicts (Fig. 5, *A* and *B*). However, the WT sequence of the full-length protein is able to form amyloid fibers along with the mutant sequences, albeit at a much slower rate (Fig. S3). This is not contradictory to our prediction, since the mutants show increased fiber-forming propensity in both contexts, but the discrepancy is interesting and warrants explanation. In the WT sequence, there exist many segments predicted to be able to form a steric zipper, inside and outside the LCD, and any of these segments could drive its amyloid formation. There may have been differences in solubility between the full-length protein and the LCD alone, especially since the LCD was conjugated to a molecule of mCherry, and if the full-length protein is less soluble, it may have been more prone to forming fibers than the LCD alone in general. In the same vein, the WT sequence of the LCD may have been able to form fibers if given more time or dissolved in different buffer conditions. Another potential explanation is the inclusion of the PB1 domain in the full-length protein, which functions as a mediator of homo-oligomerization for TFG (25). This domain, not present in the mCherry-LCD constructs, may have facilitated self-interaction of the protein, which promoted fiber formation. Regardless of the cause of the discrepancy, behavior of both the full-length protein and the LCD conjugated to mCherry was consistent with our predictions.

### Summary

In this study, we combined documented disease-causing mutations with structure-based computation to predict amyloidogenic mutations. This method was validated by the identification of known amyloidogenic mutations as well as demonstrating the formation of amyloid fibers from sequences with mutations not previously identified as amyloidogenic. Our analysis has revealed many possible unidentified amyloid proteins that need to be validated biochemically.

## Experimental procedures

### Low-complexity region prediction

Amino acid sequences in the human proteome were evaluated for low complexity using SEG reference with default settings: window length = 12, trigger complexity = 2.2, and extension complexity = 2.5. A sequence was determined to be a low-complexity region if it contained at least 35 residues scored as low complexity with at most five interrupting non–low-complexity residues.

### Protein expression and purification

Recombinant TFG (237–327) for the WT, G269V, and P285L forms was purified using a pHis-parallel-mCherry vector, using a previously described method (6). Briefly, protein was overexpressed in BL21(DE3) Gold *Escherichia coli* cells. Cultures were grown to an absorbance of 0.4 to 0.8 at 600 nm and then induced with 0.5 M IPTG overnight. Cells were pelleted by centrifugation, and the clarified lysate was purified by nickel–nitrilotriacetic acid columns followed by size-exclusion chromatography and dialyzed into PBS.

Recombinant full-length TFG was purified similarly except using a pet28b+ vector with a His tag but no mCherry and being dialyzed into buffer containing 20 mM Tris (pH 8) and 150 mM NaCl.

### Phase separation assay

Recombinant full-length TFG constructs were dissolved to 10 μM concentration in buffer containing 25 mM Tris (pH 7.4), 150 mM KCl, 2.5% v/v glycerol, and 10% w/v PEG 8000. Protein was first added to a microcentrifuge tube and diluted by adding the buffer on top of it. The total solution volume was 80 μl. Three aliquots of 20 μl were then pipetted into a Nunc 384-well clear-bottom microplate and imaged immediately using differential interference contrast microscopy.

### *In vitro* aggregation assay

WT and mutant TFG LCD was diluted to 50 μM in 1× PBS containing ThT at 40 μM to a final volume of 150 μl in black Nunc 96-well optical bottom plates (Thermo Fisher Scientific). A single polytetrafluoroethylene bead (diameter of 0.125 inch) was added to each well to facilitate agitation. Plates were incubated in a microplate reader (FLUOstar OMEGA; BMG LABTECH) for ∼138 h at 37 °C with 700 rpm double orbital shaking. Fluorescent measurements were recorded every 15 min using λ excitation = 440 nm and λ emission = 480 nm. This was performed with n = 6 technical replicates.

Aggregation assays with full-length TFG were performed with the same method, except the PBS was replaced with buffer containing 20 mM Tris (pH 8) and 150 mM NaCl.

### Transmission electron microscopy

Ten microliters of aggregated WT and mutant TFG samples (taken from *in vitro* aggregation experiments) was spotted onto carbon film on 150 mesh copper grids (Electron Microscopy Sciences) and incubated for 4 min. Grids were stained with 10 μl uranyl acetate solution (2% w/v in water) for 4 min. Excess solution was removed by blotting and air dried for 4 min. Transmission electron microscopy images were acquired with a JOEL 100CX transmission electron microscope at 100 kV.

### X-ray fiber diffraction

Aggregated samples of TFG were centrifuged at 15,000 rpm for 30 min, and buffer was exchanged with water twice. Samples were suspended between two siliconated glass capillaries ∼1 mm apart, forming a bridge between the two capillaries. Sample was allowed to dry, and the capillary with the dried aggregate was mounted on an in-house X-ray diffraction machine and diffracted with X-rays for 8 min, with the diffraction pattern collected on a charge-coupled device detector.

## Data availability

The data that support the findings of this study are available from the corresponding author, D. S. E., upon reasonable request.

## Supporting information

This article contains supporting information.

## Conflict of interest

D. S. E. is SAB Chair and equity holder in ADRx, Inc. All the other authors declare that they have no conflicts of interest with the contents of this article.
