# Supplementary material for: Bioinformatic identification of previously unrecognized amyloidogenic proteins
Source: J Biol Chem. 2022 Apr 9;298(5):101920. doi: 10.1016/j.jbc.2022.101920 (PMC9108986; doi:10.1016/j.jbc.2022.101920)
Supplement: Supplemental Tables S1–S2 and Figures S1–S5 [file mmc1.pdf]

Bioinformatic identification of previously unrecognized amyloidogenic proteins

Gregory M. Rosenberg[1], Kevin A. Murray[1], Lukasz Salwinski[1], Michael P. Hughes[1,2], Romany Abskharon[1], David S. Eisenberg[1*]

[1]Departments of Chemistry and Biochemistry and Biological Chemistry, UCLA-DOE Institute, Molecular Biology Institute, and Howard Hughes Medical Institute, UCLA, Los Angeles, CA; [2]Department of Cell and Molecular Biology, St. Jude Children's Research Hospital, Memphis, TN

*to whom correspondence should be addressed: David S. Eisenberg: University of California-Los Angeles 611 Charles E Young Drive, Boyer 201, Los Angeles, CA 90095; david@mbi.ucla.edu; Tel. (310) 825-3754, Fax. (310) 206-3914

Material included in supporting information:

- Table S1
- Table S2
- Figure S1
- Figure S2
- Figure S3
- Figure S4
- Figure S5

Table S1 Mutations in LCDs which alter a non-amyloidogenic WT segment into an amyloidogenic mutant segment. The WT and mutant scores included in the table are representative of the hexamer pair with the most dramatic negative change from the WT to the mutant score. WT and mutant scores are in units kcal/mol. The table is sorted by gene name in alphabetical order.

Table S2 Comparison of distribution of mutation types predicted to be amyloidogenic or not for each WT residue. P Values obtained through Fishers exact test. P Values could not be calculated for some initial values where 100% of the mutations were predicted to be amyloidogenic or not. Hypothesis tested is whether there is a difference between the distributions of the mutations that were predicted to be amyloidogenic versus not amyloidogenic.

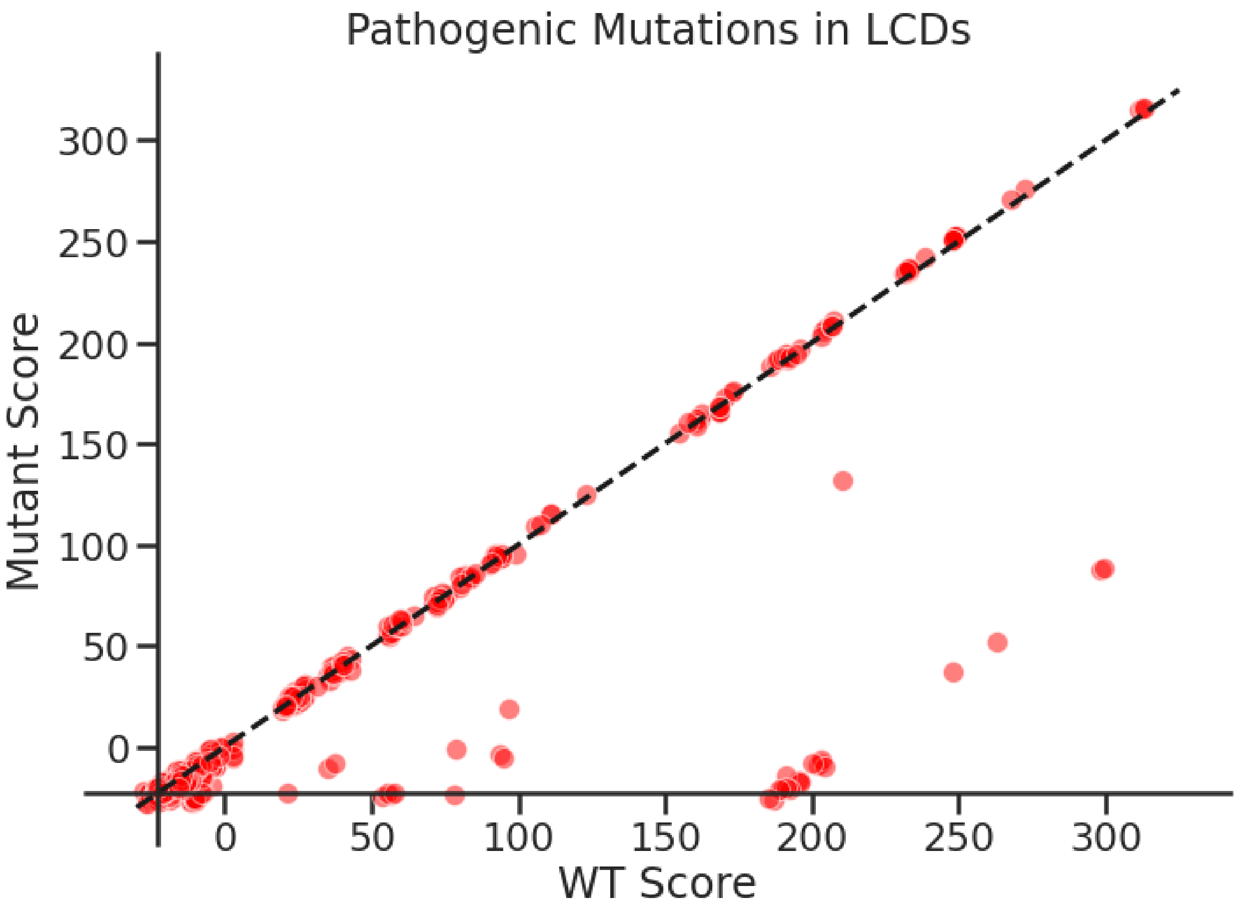

Figure S1 Full plot of ZipperDB scores for WT and mutant sequences. The vast majority of mutations with very high positive ZipperDB scores for the WT and/or mutant score are due to sequences containing prolines which are unlikely to form steric zippers.

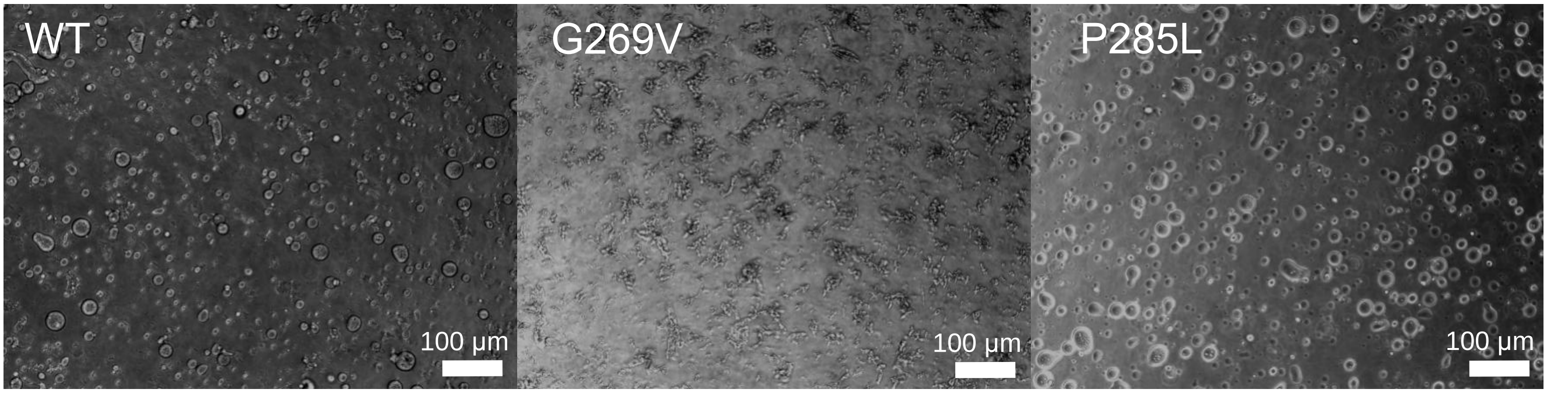

Figure S2 Phase separation properties of full length TFG mutants. Constructs were diluted to 10μM concentration in buffer containing 25mM Tris pH 7.4, 150mM KCl, 2.5% v/v glycerol, and 10% w/v PEG 8000. Differential interference contrast (DIC) microscopy images were taken at 10x magnification immediately after adding buffer to the proteins. WT and P285L separated into many large, roughly circular droplets while G269V formed amorphous aggregates.

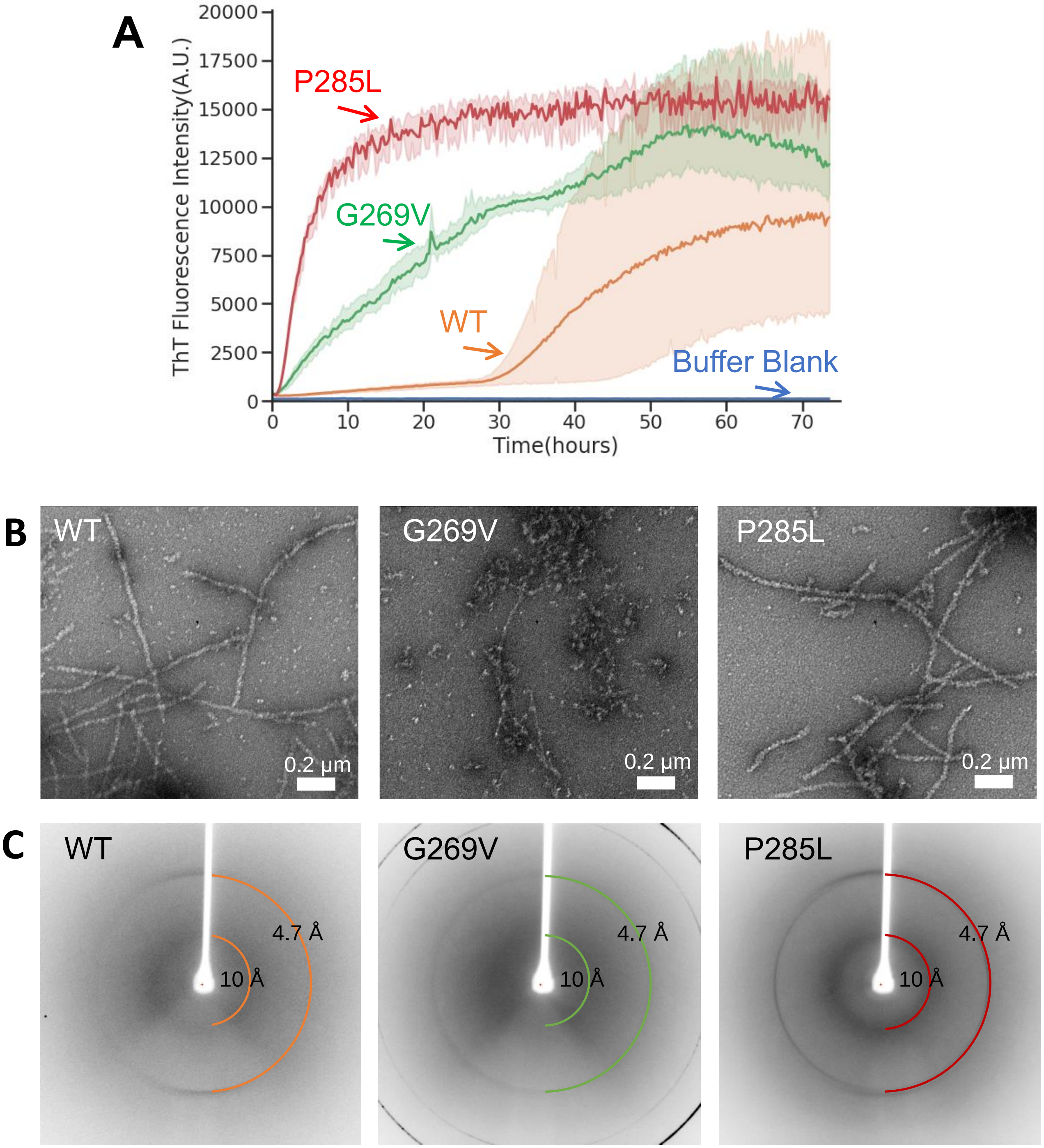

Figure S3 Amyloid properties of full-length protein TFG. A) Time dependent ThT fluorescence over time for full length TFG mutants. G269V and P285L are documented pathogenic mutations of TFG. All constructs are at 50µM concentration in buffer containing 20mM Tris pH8 and 150mM NaCl with ThT at 40µM concentration. Each construct has n=3 technical replicates and y-axis values represent the mean ThT fluorescence value of all replicates for each construct. B) Electron micrographs of the samples at the endpoint of the ThT curves. Fibers were present in all constructs. C) X-ray fiber diffraction of TFG fibers. Rings are present at 4.7 Å and 10 Å spacing with distinct wedges, indicative of cross-β structure

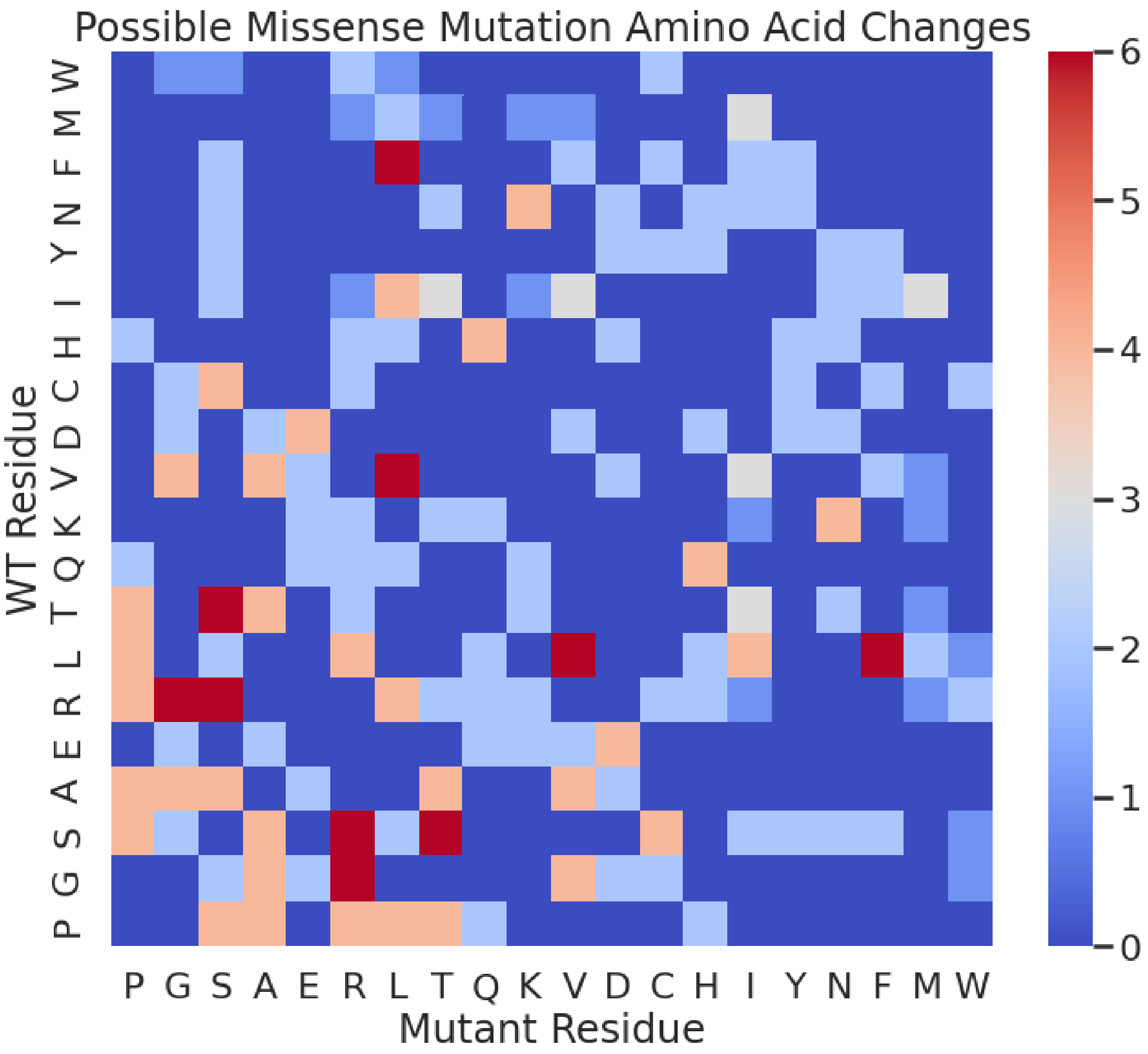

Figure S4 Counts of possible missense mutations by single nucleotide variants. The values in each cell represent the number of ways a codon for the WT residue could be mutated into a codon for the mutant residue through single nucleotide variants.

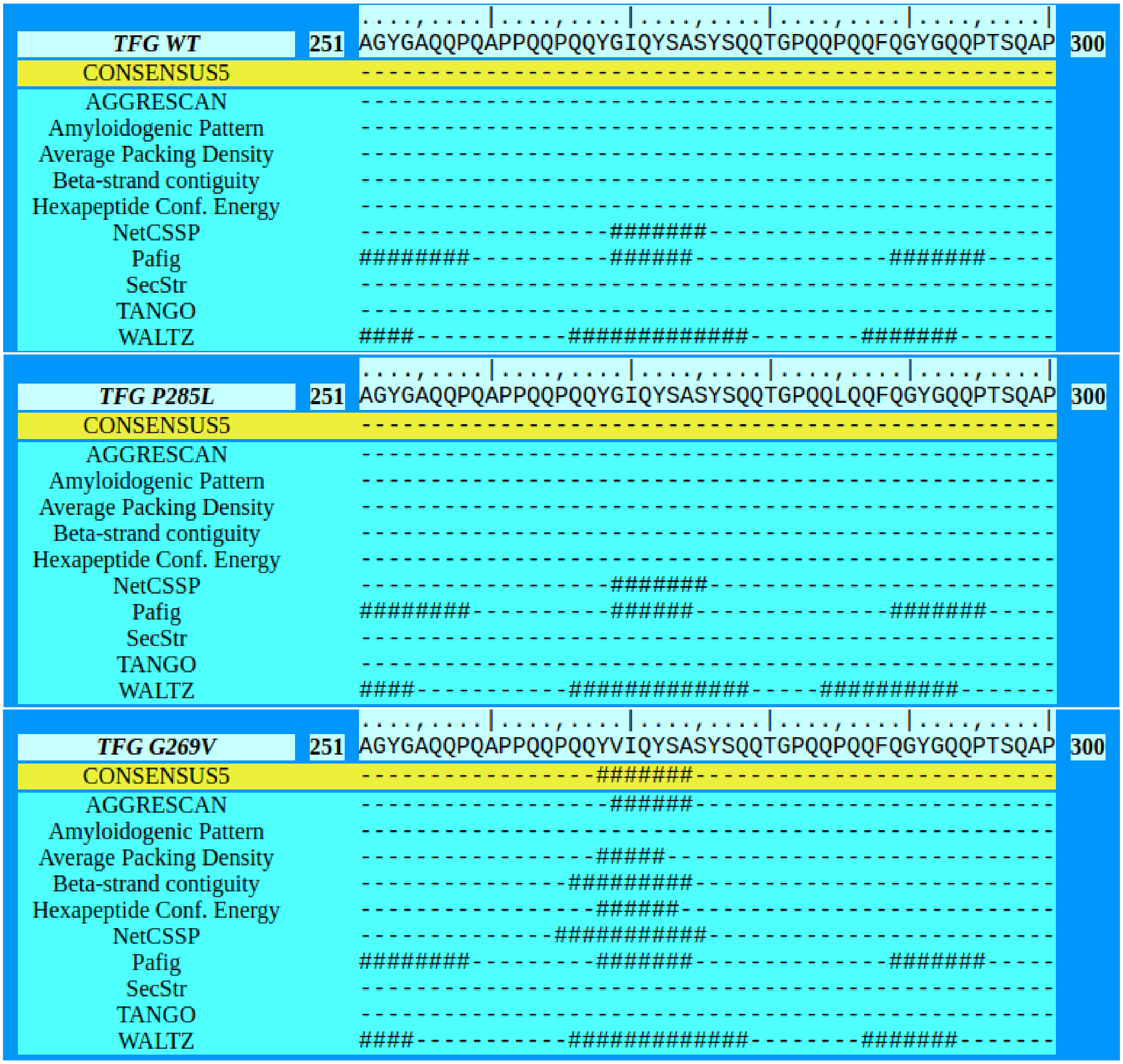

Figure S5 Output of AMYLPRED2 for amino acids 251-300 of the TFG WT sequence (top), TFG with the P285L mutation (middle), and TFG with the G269V mutation (bottom). Number signs represent positions predicted to be a part of an amyloidogenic region. The light blue box shows the predicted amyloid regions for each prediction tool incorporated into AMYLPRED2. The yellow box shows regions for which at least 5 out of the 10 prediction methods overlapped in their predicted amyloid segments. Only the G269V mutation had a consensus between enough of the amyloid predictors to cause AMYLPRED2 to have a hit in this region. The only change between the WT and P285L sequence was a slight change in the output of WALTZ with one of its predicted amyloid segments extending to include the leucine at position 285 in the mutant sequence. Images taken from http://thalis.biol.uoa.gr/AMYLPRED2/
